# Supplementary material for: Brain MRI Segmentation using Template-Based Training and Visual Perception Augmentation
Source: ArXiv. 2023 Aug 4:arXiv:2308.02363v1. Preprint. [Version 1] (PMC10418519)
Supplement: 1 [file NIHPP2308.02363V1-supplement-1.pdf]

Supplementary Figures

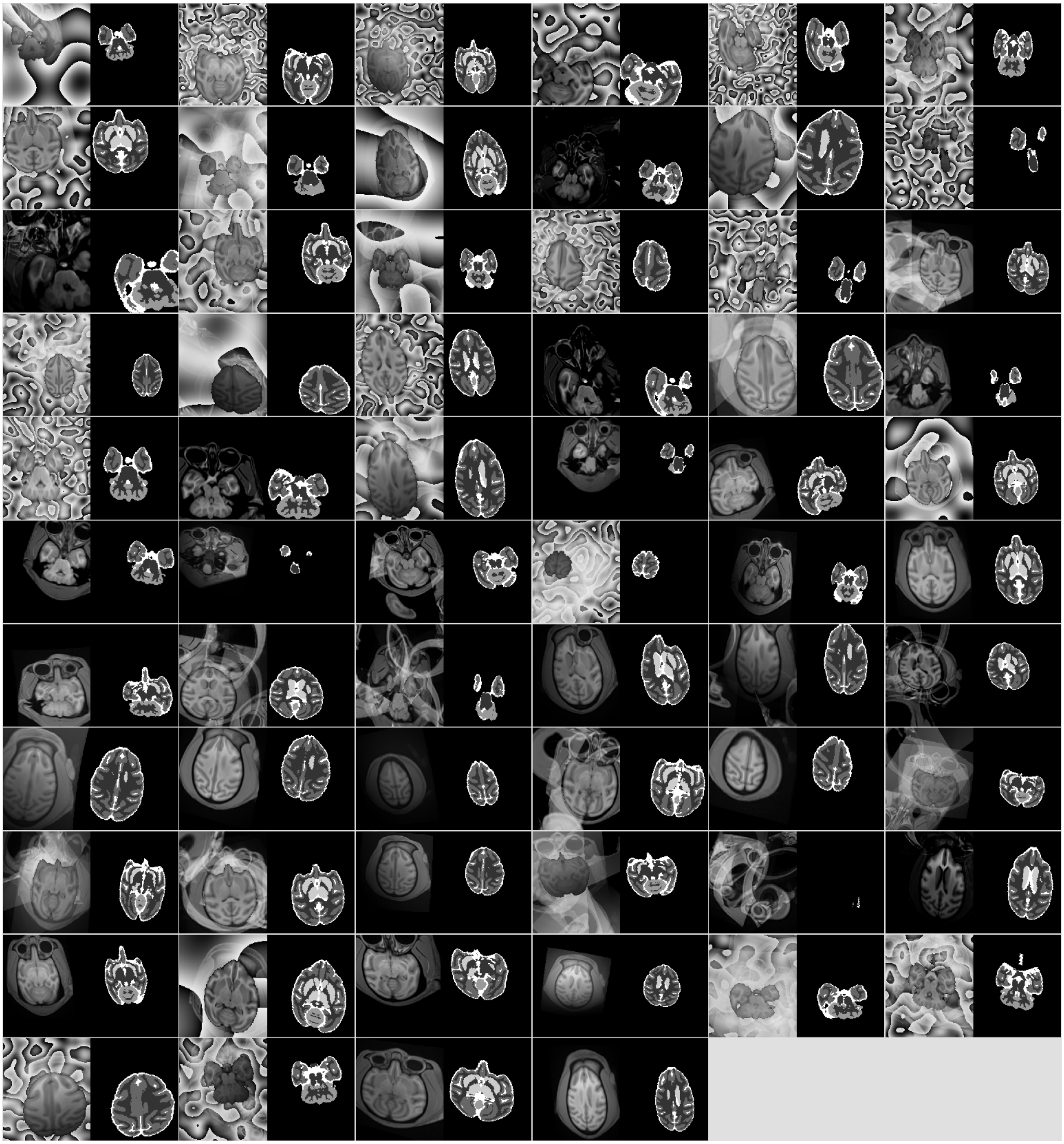

Suppl Fig. 1

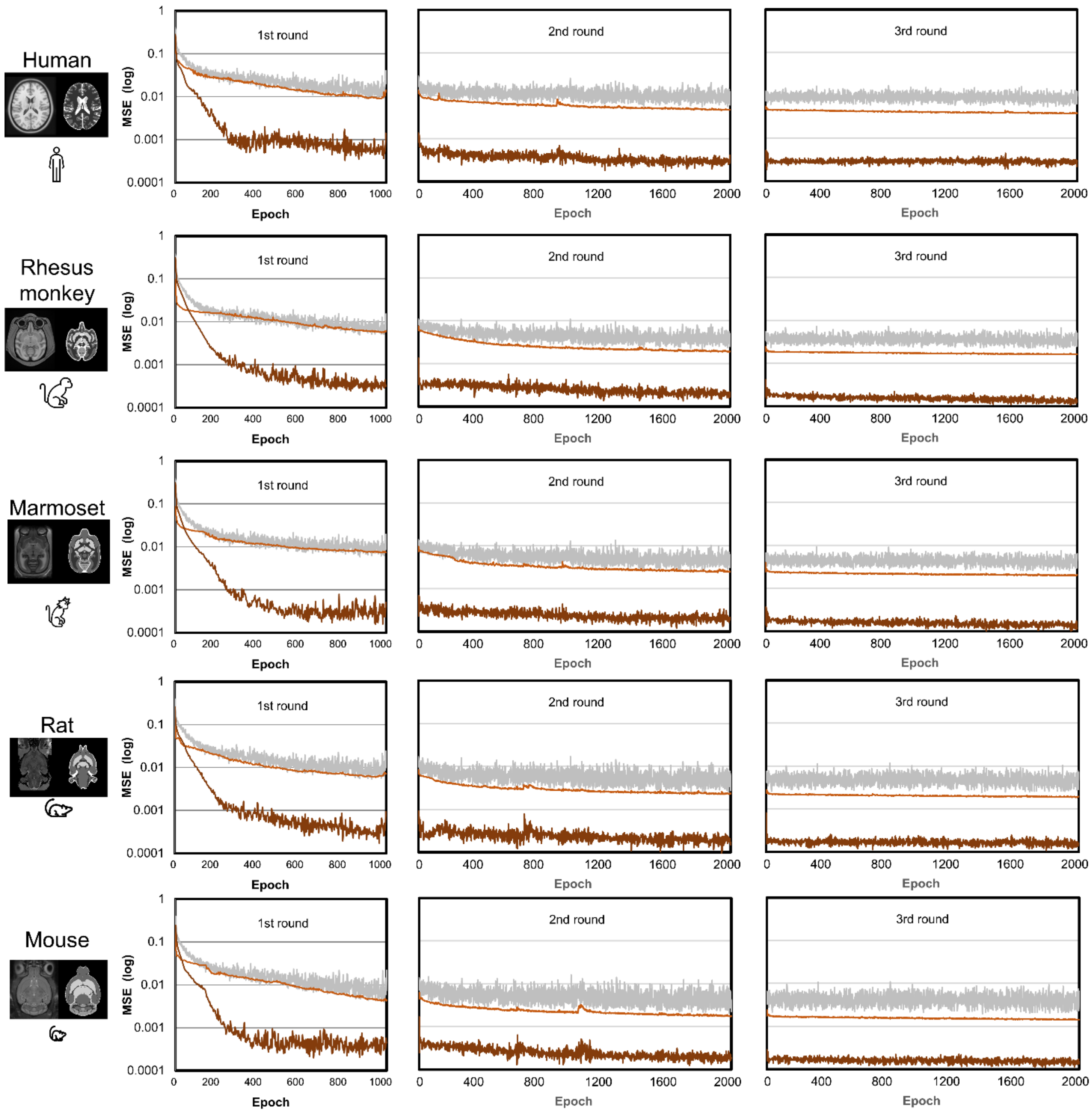
Human
Rhesus monkey
Marmoset
Rat
Mouse
1st round
2nd round
3rd round
MSE (log)
Epoch
1
0.1
0.01
0.001
0.0001
0
200
400
600
800
1000
1200
1600
2000

Suppl Fig. 2

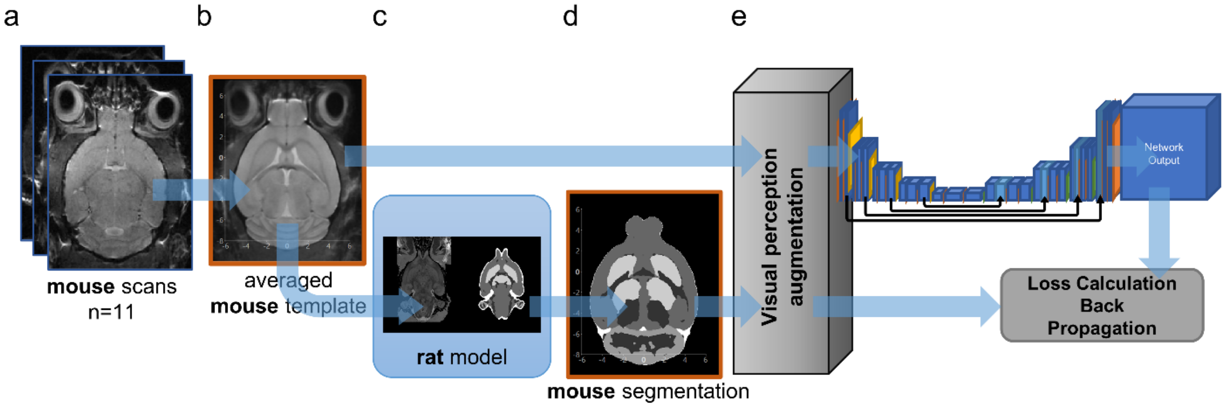
a
b
c
d
e
mouse scans
n=11
averaged
mouse template
rat model
mouse segmentation
Visual perception
augmentation
Network
Output
Loss Calculation
Back
Propagation

Suppl Fig. 3

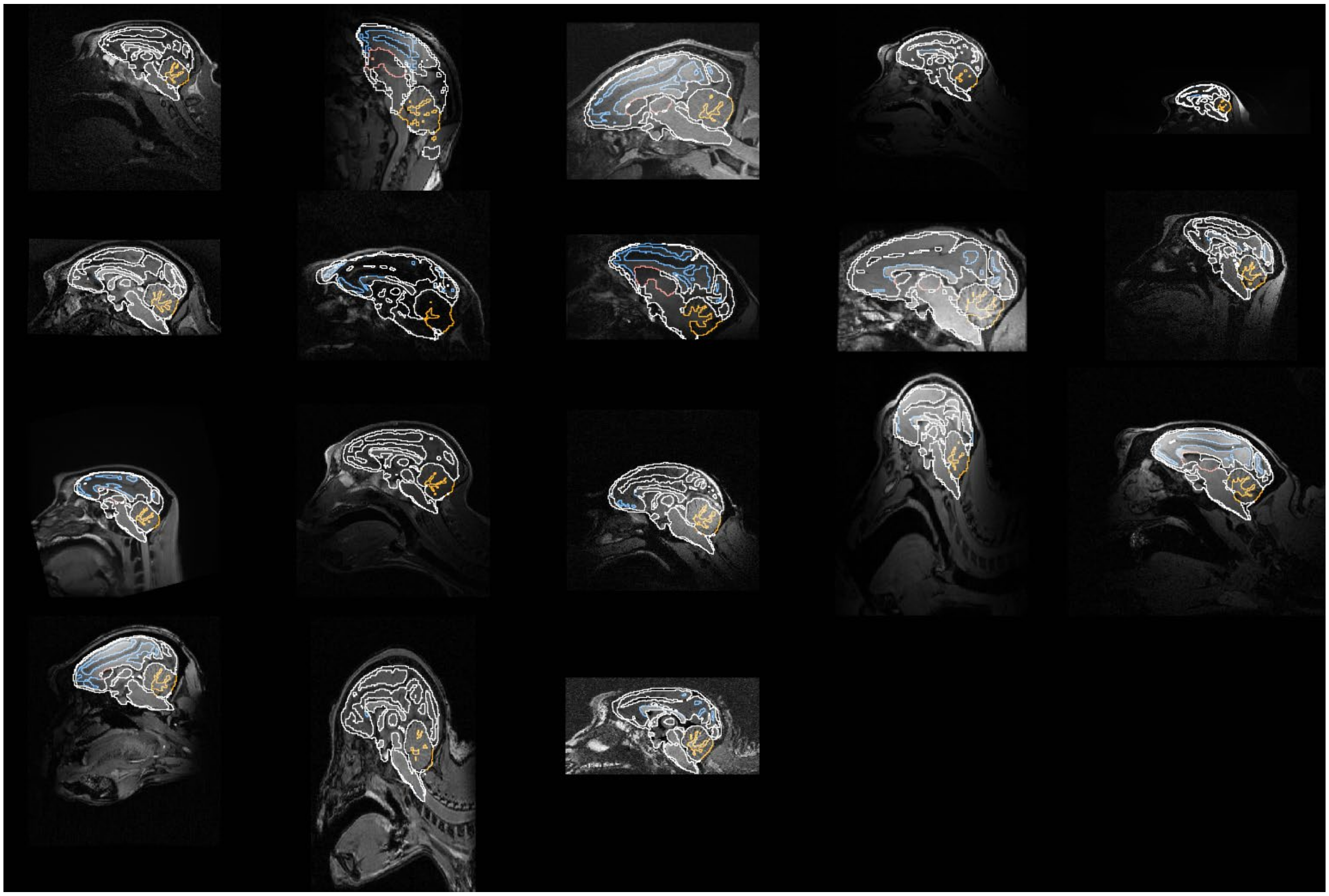

Suppl Fig. 4

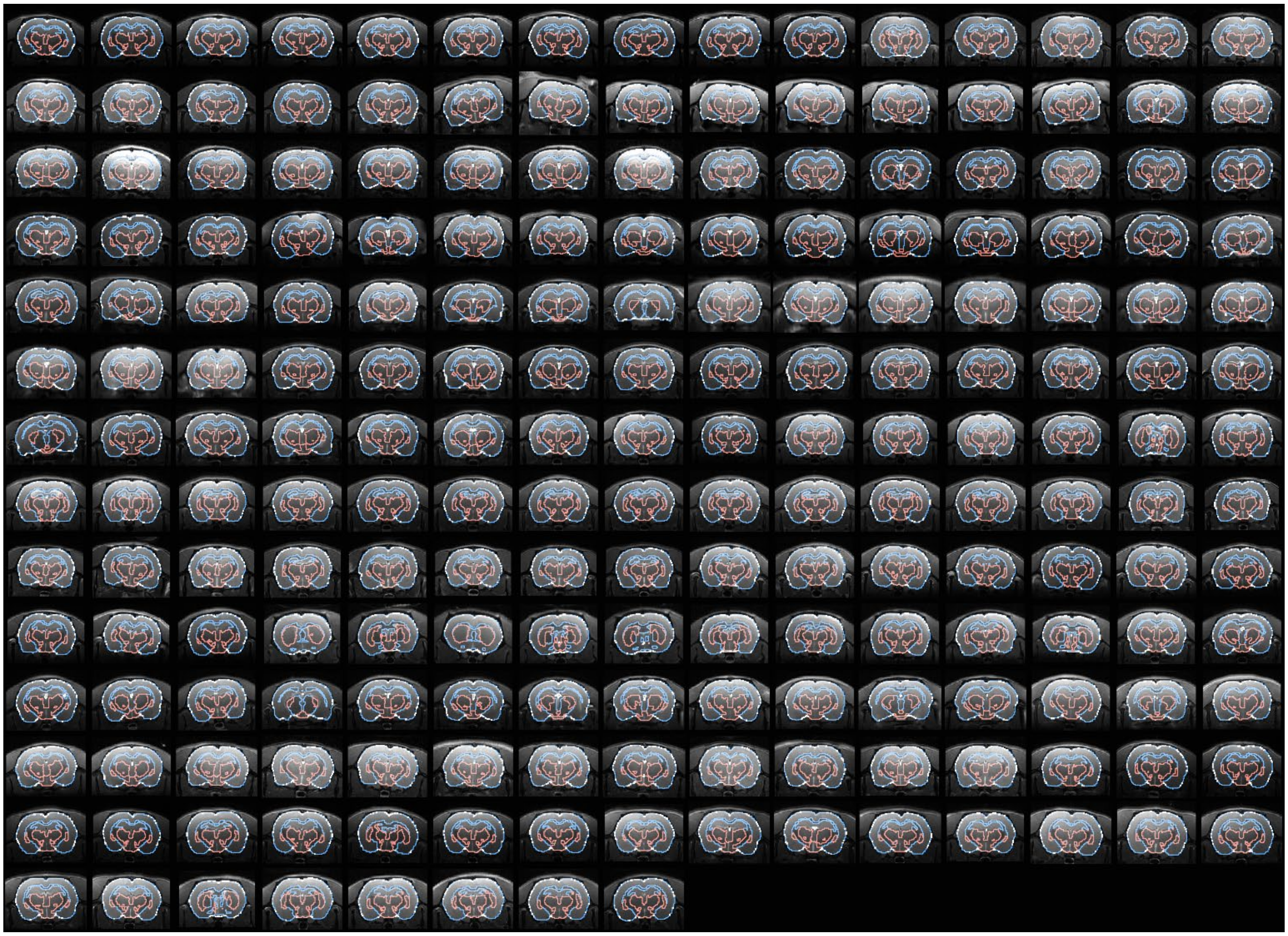

Suppl Fig. 5

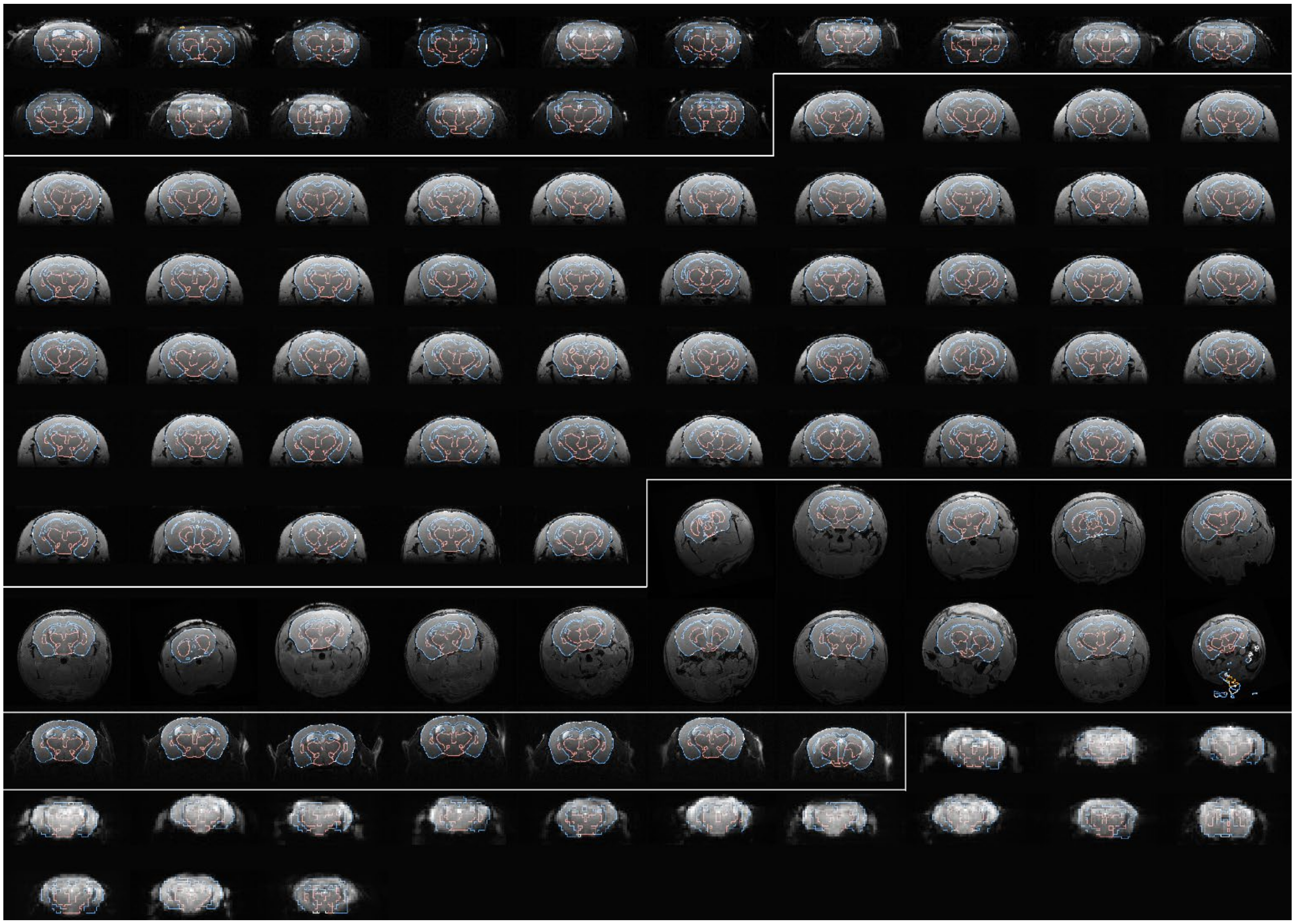

Suppl Fig. 6

Supplementary Tables

Supplementary Table 1: Source of Template Data for Visual Perception Augmentation

Species	Image Source	Label Source	Processing Applied	Label editing
**Human**	ICBM152 2009a T1W T2W [1]<br><br>https://www.bic.mni.mcgill.ca/ServicesAtlases/ICBM152NLin2009	Freesurfer applied to ICBM152 2009a T1W images	The template Image was padded to 192×224×192	Regions near brainstem, optic chiasm, and mid brain were edited on DSI Studio.
**Rhesus**	ONPRC18 Large FOV T1W T2W [2]<br><br>https://www.nitrc.org/projects/onprc18_atlas	ONPRC18 gray matter and while matter label maps.<br><br>Regions at cerebellum were edited on DSI Studio.	The template Image was padded to 192×224×192<br><br>The large FOV images were corrected by replacing images within the brain mask with the skull-stripped T1W T2W.	Regions near cerebellum were edited on DSI Studio.
**Marmoset**	Marmoset Brain Atlas V3 T1W T2W<br><br>https://marmosetbrainmapping.org/data.html	Marmoset Brain Atlas V3 T1W T2W	The template Image was padded to 192×224×192	No additional editing applied
**Rat**	The MRI data of the SIGMA rat brain template<br><br>https://www.nitrc.org/projects/sigma_template	The MRI data of the SIGMA rat brain template	The template Image was padded to 260×342×184	Regions near cerebellum were edited on DSI Studio.
**Mouse**	T2W images were nonlinearly averaged from Brookhaven National Laboratory (BNL) research<br><br>https://www.nitrc.org/projects/c57bl_mr_atlas	Generated using the rat SIGMA model	The T2W images were nonlinearly averaged from the n=11 BNL data<br><br>The template Image was padded to 288×352×224	No additional editing applied

[1] VS Fonov, AC Evans, K Botteron, CR Almli, RC McKinstry, DL Collins and BDCG, Unbiased average age-appropriate atlases for pediatric studies, NeuroImage,Volume 54, Issue 1, January 2011, ISSN 1053–8119, DOI: 10.1016/j.neuroimage.2010.07.033
[2] Weiss, Alison R., et al. "The macaque brain ONPRC18 template with combined gray and white matter labelmap for multimodal neuroimaging studies of nonhuman primates." Neuroimage 225 (2021): 117517.
[3] Liu C, et al. Marmoset Brain Mapping V3: Population multi-modal standard volumetric and surface-based templates. Neuroimage (2021) doi:10.1016/j.neuroimage.2020.117620.
[4] Barrière DA, Magalhães R, Novais A, Marques P, Selingue E, Geffroy F, Marques F, Cerqueira J, Sousa JC, Boumezbeur F, Bottlaender M. The SIGMA rat brain templates and atlases for multimodal MRI data analysis and visualization. Nature communications. 2019 Dec 13;10(1):5699.
[5] Ma, Y., et al., In Vivo 3D Digital Atlas Database of the Adult C57BL/6J Mouse Brain by Magnetic Resonance Microscopy. Front Neuroanat, 2008. 2: p. 1.

Supplementary Table 2: Source of evaluation images

Species	Source	Image Information	Scan ID	Links
**Human**	Human Connectome Project Young Adult [1]	T1W preprocessed image at 0.75-mm isotropic resampled to 1-mm isotropic	100206	https://db.humanconnectome.org/
**Chimpanzee**	National Chimpanzee Brain Resource	T1W image at 0.5-mm isotropic resolution	Agatha	https://www.chimpanzeebrain.org/
**Rhesus**	PRIMatE Data Exchange (PRIME-DE) Mount Sinai School of Medicine (Philips) [2]	T1W image at 0.5-mm isotropic resolution	032146	https://fcon_1000.projects.nitrc.org/indi/indiPRIME.html
**Marmoset**	Brain/MINDS Marmoset Brain MRI Dataset NA216 and eNA91 [3]	T1W at 0.27-mm isotropic resolution	001	https://dataportal.brainminds.jp/marmoset-mri-na216
**Rat**	Standard Rat [4]	T2-RARE at 0.2-mm isotropic resolution	105	https://openneuro.org/datasets/ds004116/versions/1.0.0
**Mouse**	GDM offsprings [5]	T2w FLASH 3D at	K6M72	https://openneuro.org/datasets/ds004145/versions/1.0.0

[1] Van Essen DC, Smith SM, Barch DM, Behrens TE, Yacoub E, Ugurbil K, Wu-Minn HCP Consortium. The WU-Minn human connectome project: an overview. Neuroimage. 2013 Oct 15;80:62-79.
[2] Milham M, Petkov CI, Margulies DS, Schroeder CE, Basso MA, Belin P, Fair DA, Fox A, Kastner S, Mars RB, Messinger A. Accelerating the evolution of nonhuman primate neuroimaging. Neuron. 2020 Feb 19;105(4):600-3.
[3] Hata J, Nakae K, Tsukada H, Woodward A, Haga Y, Iida M, Uematsu A, Seki F, Ichinohe N, Gong R, Kaneko T. Multi-modal brain magnetic resonance imaging database covering marmosets with a wide age range. Scientific Data. 2023 Apr 27;10(1):221.
[4] Grandjean J, Desrosiers-Gregoire G, Anckaerts C, Angeles-Valdez D, Ayad F, Barrière DA, Blockx I, Bortel A, Broadwater M, Cardoso BM, Célestine M. A consensus protocol for functional connectivity analysis in the rat brain. Nature neuroscience. 2023 Mar 27:1-9.
[5] Xin Yi Yeo and HanGyu Bae and Ling-Yun Yeow and Hongyu Li and Li Yang Tan and Woo Ri Chae and Joanes Grandjean and Weiping Han and Sangyong Jung (2022). GDMOffspring_MRI. OpenNeuro. [Dataset] doi: doi:10.18112/openneuro.ds004145.v1.0.0

Supplementary Table 3: Blind evaluation results

Evaluator Votes (Sorted)	A versus B		A versus C		B versus C		Evaluator background	
	A>B	B>A	A>C	C>A	B>C	C>B	PhD	MD
A=CB	1					1	x	
A=CB	1					1		
AB=C	1		1				x	
AB=C	1		1					x
AB=C	1		1					x
ABC	1		1		1		x	
ABC	1		1		1		x	
ABC	1		1		1		x	x
ACB	1		1			1	x	
ACB	1		1			1	x	
ACB	1		1			1	x	
ACB/CBA	0.5	0.5	0.5	0.5		1		x
BCA		1		1	1			
BCA		1		1	1			x
CA=B				1		1	x	
CA=B				1		1	x	
CAB	1			1		1	x	x
CAB	1			1		1	x	
CAB	1			1		1	x	
CAB	1			1		1	x	
CAB	1			1		1		
CAB	1			1		1	x	
CAB	1			1		1	x	
CAB	1			1		1	x	
CAB	1			1		1	x	
CAB	1			1		1		
CAB	1			1		1	x	
CAB	1			1		1		x
CAB	1			1		1		
CBA		1		1		1	x	
CBA		1		1		1	x	
CBA		1		1		1	x	
CBA		1		1		1		x
CBA		1		1		1	x	
CBA		1		1		1		
	24.5	8.5	9.5	23.5	5	27	23	8
